# Fuzheng Yiliu Formula Regulates Tumor Invasion and Metastasis through Inhibition of WAVE3 Expression

**DOI:** 10.1155/2021/8898668

**Published:** 2021-03-27

**Authors:** Wen-li Chen, Huan-huan Bai, Li-wei Liu, Hong-yu Chen, Qi Shi, Li-sheng Chang, Xiao-jun Gou, Jun Qian

**Affiliations:** ^1^Baoshan District Hospital of Integrated Traditional Chinese and Western Medicine of Shanghai, Shanghai 201999, China; ^2^School of Pharmacy, Shaanxi University of Traditional Chinese Medicine, Xianyang, Shaanxi 712046, China; ^3^Department of Diagnostics of Chinese Medicine, School of Chinese Medicine, School of Integrated Chinese and Western Medicine, Nanjing University of Chinese Medicine, Nanjing, Jiangsu 210023, China

## Abstract

**Objective:**

To explore the mechanism of action of Fuzheng Yiliu formula (FZYLF) in regulation of the invasion and metastasis of MDA-MB-231/Adr human breast cancer cells through WAVE3.

**Methods:**

The MDA-MB-231/Adr cells with high invasive ability were screened by Transwell, and the plasmid with high WAVE3 expression was made for transfection. Plasmid transfection efficiency and protein expression level were verified by polymerase chain reaction (PCR) and western blotting (WB). The effect of FZYLF on cell proliferation and invasion was investigated before and after WAVE3 silencing by flow cytometry. A nude mouse model of tumor metastasis was established to study the antitumor activity of FZYLF.

**Results:**

The expression levels of mRNA and proteins of intracellular WAVE3 increased significantly after plasmid transfection, mRNA from 1.37± 0.41 to 9.88 ± 1.31 and protein from 1 ± 0.08 to 5.09 ± 0.03 (*P* < 0.01). Intervention with FZYLF could significantly affect the activity of MDA-MB-231/Adr cells and inhibit invasion and metastasis, IC_50_ from 71.04 to 46.41 mg/mL and from 162 ± 14.82 to 81.4 ± 12.05 (*P* < 0.05 or *P* < 0.01), and significantly reduce the expression levels of WAVE3 (from 1 ± 0.02 to 0.63 ± 0.04), MMP-9 (from 1 ± 0.05 to 0.63 ± 0.03), NF-*κ*B (p65) (from 1 ± 0.02 to 0.62 ± 0.02), and p-I*κ*B*α* (from 1 ± 0.03 to 0.68 ± 0.02) (*P* < 0.05 or *P* < 0.01). The T/C (%) of FZYLF (13 g crude drug/kg) was 62.06% for MDA-MB-231/Adr tumor xenografted in nude mice, with a tumor inhibition rate of 39.64%.

**Conclusion:**

FZYLF can inhibit the invasion and proliferation of the MDA-MB-231/Adr human breast cancer cells, and the mechanism of action may be related to the regulation of WAVE3 expression.

## 1. Introduction

Breast cancer is one of the most common malignant tumors in women all over the world and has gradually evolved into the first female malignant tumor in recent years [1]. This disease tends to recur and metastasize and its biological behavior is dangerous, especially in triple negative breast cancer (TNBC), which has the characteristics of poor histological grade, advanced clinical stage, poor prognosis and high mortality, and a tendency to occur at a younger age. The 3- and 5-year disease-free survival (DFS) and overall survival (OS) are lower in TNBC than in non-TNBC [2]. The prognosis of TNBC gets worse with the increase in N staging, and the probability of recurrence and metastasis increases significantly at stage N2 or above, but there is no significant correlation with T staging and histological grade [3]. It is estimated that up to 30% of patients with lymph node negative breast cancer and most patients with lymph node positive breast cancer will have metastasis even with standardized treatment. With the continuous application of chemotherapy drugs, chemotherapy-induced multidrug resistance (MDR) of breast cancer not only limits the clinical efficacy of drugs but also promotes the occurrence of invasion and metastasis. The mechanism of MDR development is complex in breast cancer and is often believed to be the combined result of multiple mechanisms, including high expression of membrane transporters, disorders in drug transport and activation, changes in the nature and number of drug targets, changes in the enzyme system, enhancement of DNA repair, and inhibition of cell apoptosis [4].

WAVE3 plays an important role in migration and invasion of breast cancer cells. Breast cancer cells degrade the extracellular matrix and basement membrane at the primary site, migrate into blood vessels, and reach the distal organs through blood flow to form metastatic foci [5]. WAVE3 (also known as WASF3) is a member of the WASP (Wiskott–Aldrich syndrome) protein family, which is a newly discovered group of proteins in recent years that mediates actin polymerization by activating actin-related protein 2/3 (Arp2/3) complex and participates in the formation of filamentous pseudopodia and lamellar pseudopodia during cell migration, membrane transport, and cell adhesion. WAVE3 plays a critical role in tumor proliferation, differentiation, and invasion, especially in invasion and metastasis. In breast cancer, the expression of WAVE3 is positively correlated with the grade, drug resistance, invasion, and progression of tumor. In TNBC, the high expression of WAVE3 is closely related to the metastasis of breast cancer [6]. WAVE3 is also an important link in TGF*β*-induced epithelial matrix transformation in breast cancer [7]. Silencing the expression of WAVE3 in invasive breast cancer cells by siRNA can effectively reduce the number of lamellar pseudopodia on the edge of tumor cell membrane and weaken the ability of tumor cells to invade and migrate. After the breast cancer cells with further knockout of WAVE3 are inoculated into nude mice, it is found out that compared to the negative control group, the growth of tumor *in situ* is significantly slower and the distant metastasis rate of tumor decreases significantly [8]. In addition, WAVE3 knockout also increases the sensitivity of TNBC to chemotherapy by inhibiting STAT-HIF-1*α*-mediated angiogenesis [9]. In our recent study, it has been confirmed that the expression of WAVE3 is also higher in tissues of invasive breast cancer than in tissues of breast cancer *in situ* [10]. These results suggest that WAVE3 plays an important role in the invasion and metastasis of breast cancer, especially in chemotherapy-resistant breast cancer.

Matrix metalloproteinase-9 (MMP-9) is a secreted multidomain enzyme that regulates the composition of cell matrix. It belongs to the gelatinase subfamily of MMPs. Therefore, its main substrate is gelatin (collagen), which is the main component of basement membrane. MMP-9 destroys the basement membrane and promotes tumor invasion and metastasis by enzymolysis. Christine et al. have found that MMP-9 secreted by human breast cancer cells promotes the invasion of tumor cells in cell culture and lung metastasis *in situ* mouse model of basal-like TNBC [11]. Zhao et al. have revealed that in TNBC, prognosis is worse in patients with high expression of MMP-9 than in patients with low expression of MMP-9 [12].

I*κ*B*α* is the most classic member of I*κ*B protein family. The NF-*κ*B family is composed of five members, namely, P50, P52, P65, c-Rel, and RelB. In the classic NF-*κ*B signaling pathway, I*κ*B*α* binds with the most common NF-*κ*B dimer P65 : P50 to prevent it from entering the nucleus and binding with DNA, thus inhibiting the activation of NF-*κ*B signaling pathway. When I*κ*Ba is phosphorylated by activated IKK to p-I*κ*B*α*, the classic NF-*κ*B signaling pathway is activated. NF-*κ*B plays an important role in the occurrence and development of breast cancer. Hossain et al. have found out that Notch signal regulates mitochondrial metabolism and NF-*κ*B activity in TNBC cells through IKK*α*-dependent nonclassical pathway, thus promoting the invasion and metastasis of breast cancer [13]. El-Hafeez et al. have revealed that norwogonin can further inhibit the activation of NF-*κ*B and STAT3 signaling pathways by inhibiting the expression of TAK1, thus playing an anticancer role [14].

Traditional Chinese medicine (TCM) has unique advantages in preventing tumor recurrence and metastasis [15, 16]. A study by Zheng et al. [4] has suggested several reversal strategies of MDR in breast cancer, including reversal by chemical drugs, cytokines, immunotherapy, gene technology, and Chinese medicines, and treatment with TCM compound formulas has the advantages of multitarget, safety, effectiveness, and low cost. Fuzheng Yiliu formula (FZYLF, formula that reinforces healthy qi and inhibits tumor) is a commonly used prescription in our department that has been used over 10 years in patients after operation for breast cancer. It is composed of Dangshen (Radix Codonopsis, DS), Maidong (Radix Ophiopogonis, MD), Maorenshen (*Actinidia valvata* Dunn, MRS), Shijianchuan (Salviae Chinensis Herba, SJC), stir-fried Biejia (Carapax Trionycis, BJ), crude Yiyiren (Semen Coicis, YYR) (DS 15 g, MD 10 g, MRS 15 g, SJC 15 g, BJ 15 g, and YYR 15 g), and so on. In this prescription, DS tonifies qi and engenders fluid; MD nourishes yin and moistens the lung; stir-fried BJ softens the lumps and dissipates the nodules; MRS clears heat and removes toxin; SJC clears heat and removes toxin, activates blood, and suppresses pain; crude YYR invigorates the spleen and drains dampness, eliminates impediment and checks diarrhea, clears heat, and expels pus. These drugs used in combination strengthen healthy qi and inhibit tumor. Previous clinical studies have confirmed that this prescription can significantly improve the patients' quality of life [17, 18]. From the perspective of basic research, the present study discusses the effect and mechanism of FZYLF on the invasion and metastasis of multidrug-resistant breast cancer cells.

## 2. Materials and Methods

### 2.1. Experimental Materials

#### 2.1.1. Cell Lines

The MCF-7 and MDA-MB-231 human breast cancer cells and the drug-resistant cell lines MCF-7/Taxol, MCF-7/Adr, MDA-MB-231/Taxol, and MDA-MB-231/Adr were cultured in DMEM medium containing 10% fetal bovine serum (FBS) at 37°C with 5% CO_2_.

#### 2.1.2. Animals

25 female SPF BALB/c nude mice were provided by Changzhou Cavens Laboratory Animal Co., Ltd. (Changzhou, China) (laboratory animal production license: SCXK (SU) 2016-0010; laboratory animal use license: SYXK (SU) 2017-0007).

#### 2.1.3. Reagents and Consumables

DMEM (high sugar) medium was purchased from Gibco (NY, USA); FBS was obtained from ScienCell Research Laboratories (CA, USA). CCK8 cell viability test kit was purchased from Enjing Biotech Co., Ltd. (Nanjing, China) (E1CK-000208-10). Transwell chamber with pore size of 8.0 *μ*m was manufactured by Corning Incorporated (REF 3422, LOT: 14416045). BD Matrigel matrix (basement membrane) was purchased from BD Biosciences (356234). WAVE3 antibody was from EnoGene, rabbit antihuman/mouse (E20-74899); I*κ*B-*α*antibody from EnoGene, rabbit antihuman/mouse (E2344534); P-I*κ*B-*α*antibody from EnoGene, rabbit antihuman/mouse (E2340776); NF-*κ*B antibody from EnoGene, rabbit antihuman (E10-20406); MMP-9 antibody from EnoGene, rabbit antihuman (E11-0275C); GAPDH antibody from EnoGene, rabbit antihuman/mouse (E90062). Hydrophobic PVDF membrane was obtained from Millipore; ECL chemiluminescence reagent was obtained from Beyotime (P0018A). RIPA Lysis solution, BSA, prestain protein marker, HRP labeled goat anti-rabbit secondary antibody, developer, fixer, and BCA protein assay kit were all purchased from Enjing Biotech Co., Ltd. (Nanjing, China). WAVE3 plasmid was purchased from Nanjing GenScript Biotechnology Co., Ltd. (Nanjing, China).

MRS (Lot no. 160108) in FZYLF was grown in Zhejiang Province, produced on January 08, 2016, and processed by Hongqiao Chinese Medicine Crude Slices Co., Ltd. (Shanghai, China). Other medicinal materials (Lot no. 1512068) were grown in Zhejiang Province and processed by Shanghai Lei Yunshang Chinese Medicine Crude Slices Factory (Shanghai, China). Amount of the drugs used in the prescription was as follows: DS 15 g, MD 10 g, MRS 15 g, SJC 15 g, stir-fried BJ 15 g (decocted first), and YYR 15 g.

#### 2.1.4. Instruments

The instruments used in this study are shown in Table 1.

#### 2.1.5. Preparation of FZYLF

3 bags of the abovementioned herbs were taken in an appropriate amount. Powder of the herbs was mixed, added with 10 times water, and soaked overnight. Reflux extraction was carried out for 2 h. The extract was recovered and filtered, then evaporated and concentrated in an evaporating dish on a water bath, and the water extract of FZYLF was obtained. The extract was concentrated to 200 mL at a concentration of 1.5 g crude drugs/mL and stored at 4°C. Quality control of Fuzheng Yiliu formula can be seen in Supplementary Materials.

### 2.2. Methods

#### 2.2.1. Effect of FZYL on Proliferation of MDA-MB-231 Human Breast Cancer Cells In Vitro

Tumor cells were cultured separately. Cells in logarithmic growth phase were inoculated into a 96-well plate at 1 × 10^5^ cells/mL, 100 *μ*L/well, and cultured for 24 h at 37°C, 5% CO_2_. Water and alcohol extracts at corresponding concentrations were added, and a negative control group was set up. After incubation with cells for 24 h, the growth of cells in each group was observed under a microscope. 10 *μ*L CCK-8 was added to each well and let stand at room temperature for 4 h. Absorbance was detected at 450 nm and IC_50_ was calculated.

#### 2.2.2. Detection of WAVE3 Expression Level

The expression of WAVE3 gene in human breast cancer cell lines MCF-7 and MDA-MB-231 and drug-resistant cell lines MCF-7/Taxol, MCF-7/Adr, MDA-MB-231/Taxol, and MDA-MB-231/Adr was detected by real-time PCR. In the previous experiment on asiatic acid, the results showed that asiatic acid could significantly inhibit proliferation in the MDA-MB-231 and MCF-7 cell lines, with the strongest inhibitory activity on MDA-MB-231. Therefore, MDA-MB-231 cells were selected for follow-up study [10].

Real-time PCR: cells were lysed in TriPure Regent at 1 mL per well in a 6-well plate. The lysate was extracted with chloroform, precipitated with isopropanol, and washed with 75% ethanol to precipitate total RNA. Total RNA was reverse-transcribed into cDNA following instructions of the reverse transcription kit, and the expression of each target gene was detected by real-time quantitative PCR with *β*-actin as internal reference. SYBR green real-time PCR was used for amplification, and the threshold value and Ct value were obtained automatically by software. The specific operation is shown in the literature [10]. The sequences of primers were used as follows: *β*-actin (5′-AGTCCTGTGGCATCCACGAAAC-3′ and 5′-CACACGGAGTACTTGCGCTCAG-3′) and WAVE3 (5′-CACCAATCAGTGATGCTCGAAG-3′ and 5′-AGTCGGACCAGTCGTTCTCG-3′).

#### 2.2.3. Expression of WAVE3 at Gene and Protein Levels after Transfection

MDA-MB-231/Adr cells were transfected with shRNA-WAVE3, and pcDNA3.1-WAVE3 was transfected into MDA-MB-231 cells. The MDA-MB-231/Adr and MDA-MB-231 cells in logarithmic growth phase were inoculated into a 6-well plate and cultured in an incubator until a cell density of 60∼80% at the bottom of the well. The cells were ready for transfection. Before transfection, the culture medium in the wells was replaced by 1 mL antibiotics-free DMEM medium. Following Lipo2000 operation instructions, 200 *μ*L each of opti-MEM was used to dilute shRNA-WAVE3 (50 nmol) or pcDNA3.1-WAVE3 and Lipo2000 (5 *μ*L), and a negative control group was set up. Cells in the control group were transfected as mentioned above with blank vector plasmid containing no WAVE3 gene. The diluted Lipo2000 was incubated for 5 min at room temperature and then gently mixed with the diluted shRNA and let it stand for 20 min at room temperature to form the shRNA-RNAiMAX complex, which was added into the cell culture plate and mixed by shaking gently. The plate was put into an incubator for incubation at 37°C, 5% CO_2_. The DMEM complete medium was replaced 6 h later.

Gene and protein expression levels of WAVE3 in the human breast cancer cell line MDA-MB-231 and the drug-resistant cell line MDA-MB-231/Adr after transfection were detected by real-time PCR and western blotting. Protein expression levels of WAVE3, I*κ*B*α*, NF-ΚB, and MMP-9 in the human breast cancer cell line MDA-MB-231/Adr shRNA after transfection were investigated by western blotting. The real-time PCR is the same as mentioned in Section 2.2.2, and the specific operation of western blot is shown in the literature [10]. In the previous experiment on asiatic acid in FZYLF, the expression of WAVE3 increased significantly in ductal carcinoma in situ tissue, and it was even higher in the metastasis group. WAVE3 might be involved in drug resistance, invasion, and metastasis of tumor cells. Therefore, MDA-MB-231/Adr cells were selected for follow-up study [10].

#### 2.2.4. Detection of Cell Proliferation, Metastasis, and Invasion after Transfection

The transfected cells were inoculated into a 96-well plate and treated with FZYLF at different concentrations (100 mg crude drugs/mL, 50 mg crude drugs/mL, 25 mg crude drugs/mL, 12.5 mg crude drugs/mL, 6.25 mg crude drugs/mL, 3.125 mg crude drugs/mL, 1.56 mg crude drugs/mL, and 0.78 mg crude drugs/mL) on the next day. CCK-8 was added 72 h later to determine OD450 and calculate the inhibition rate and IC_50_. Effect of FZYLF on in vitro cell proliferation activity, metastasis, and invasion of the human breast cancer cell line MDA-MB-231 and the drug-resistant cell line MDA-MB-231/Adr after transfection was investigated with CCK-8 kit and by Transwell assay.

Transwell method: the Transwell chamber coated with matrix gel was put into a culture plate; 300 *μ*L prewarmed serum-free medium was added into the upper chamber and kept for 15∼30 min at room temperature to rehydrate the matrix gel. The residual culture medium was removed. The cells were starved for 12 h and resuspended in serum-free medium containing BSA to make cell suspension. The cell density was adjusted to 1 × 10^5^ cells/mL. 100 *μ*L cell suspension was inoculated into the Transwell chamber and 500 *μ*L of a medium containing FBS was added into the lower chamber for routine culture for 12∼48 h. The matrix gel and cells in the upper chamber were wiped off with cotton swabs. The cells were stained with 0.1% crystal violet, and the number of cells passing through the membrane was counted under a microscope. The cells were decolorized with 33% acetic acid for complete elution of crystal violet. The eluent was collected and OD value was determined at 570 nm to indirectly reflect the number of cells.

#### 2.2.5. Model Establishment and Administration

The cell lines MDA-MB-231/Adr-siC in logarithmic growth phase and MDA-MB-231/Adr-si WAVE3 with low expression of WAVE3 were used for the experiment. Cell suspension was inoculated subcutaneously under the right armpit of nude mice at 5 × 10^6^ cells/mice under sterile conditions. Diameter of the xenografted tumor was measured with a Vernier caliper. When the tumor grew to about 100 mm^3^, the tumor-bearing nude mice that grew well and showed uniformity in tumor size were selected and divided into four groups.

Group assignment: MDA-MB-231/Adr-siC xenografted tumor group, MDA-MB-231/Adr-siC xenografted tumor + FZYLF (13 g crude drugs/kg) group, MDA-MB-231/Adr-siWAVE3 xenografted tumor group, and MDA-MB-231/Adr-siWAVE3 xenografted tumor + FZYLF (13 g crude drugs/kg) group.

FZYLF was administered by gavage to mice in each group, once daily at a dosing volume of 0.1 mL/10 g body weight. The antitumor effect of the test article was observed dynamically by measuring the tumor diameter every other day. Body weight of the mice was measured while measuring tumor diameter. The mice were sacrificed at day 15. The tumor mass was surgically removed, weighted, and preserved in formalin.

Tumor volume (TV) was calculated as follows:

(1) $$\begin{array}{c} \text{TV}=\frac{1}{2}\times a\times b^{2}, \end{array}$$

where *a* and *b* represent the length and width, respectively.

Relative tumor volume (RTV) was calculated based on the measurement results using the following formula: RTV = *V*_*t*_/*V*_0_, where *V*_0_ is the tumor volume measured at dosing at cage assignment (*d*_0_) and *V*_*t*_ is the tumor volume at each measurement. Antitumor activity was evaluated by relative tumor proliferation rate T/C (%) which was calculated as follows:

(2) $$\begin{array}{c} \frac{T}{C}\left(\%\right)=\frac{{\text{T}}_{\text{RTV}}}{{\text{C}}_{\text{RTV}}}\times 100, \end{array}$$

where T_RTV_ is RTV in the treatment groups; C_RTV_ is RTV in the negative control groups.

#### 2.2.6. Statistical Analysis

Data were expressed as mean ± SD. The GraphPad Prism 5.0 software was used for statistical analysis. T-test was performed for comparison between two groups. *P* < 0.05 indicated statistical significance.

## 3. Results

### 3.1. Effect of FZYLF on Proliferation of the Human Breast Cancer Cell Line MDA-MB-231

**Figure 1:**
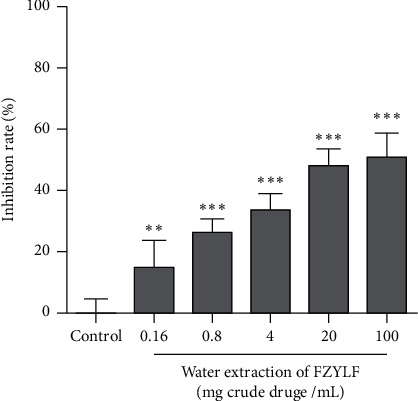
Effect of FZYLF on in vitro proliferation activity of the MDA-MB-231 human breast cancer cells. Compared with the control group, ^*∗*^*P* < 0.05, ^*∗∗*^*P* < 0.01, and ^*∗∗∗*^*P* < 0.001 (mean ± SD, *n* = 5).

Results (Figure 1) showed that FZYLF significantly inhibited proliferation of the human breast cancer cell line MDA-MB-231 (*P* < 0.01, or *P* < 0.001).

### 3.2. Gene Expression Level of WAVE3 in the Tumor Cells MCF-7, MCF-7/Taxol, MCF-7/Adr, MDA-MB-231, MDA-MB-231/Adr, and MDA-MB-231/Taxol

**Figure 2:**
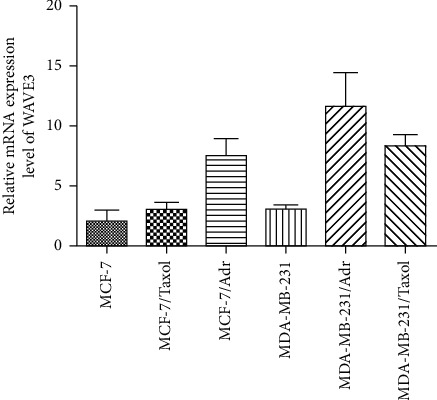
Relative expression level of WAVE3 gene in the tumor cells.

Results (Figure 2) showed that the expression level of WAVE3 was higher in the drug-resistant MDA-MB-231/Adr and MDA-MB-231/Taxol cells than in the nondrug-resistant MDA-MB-231 cells, and it was higher in the drug-resistant MCF-7/Adr and MCF-7/Taxol cells than in the nondrug-resistant MCF-7 cells. The expression level of WAVE3 was the highest in the MDA-MB-231/Adr cells.

### 3.3. mRNA and Protein Expression Levels of WAVE3 in the MDA-MB-231 and MDA-MB-231/Adr Cells after Transfection

**Figure 3:**
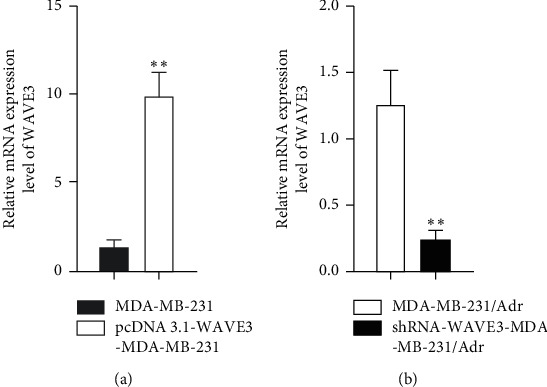
mRNA expression level of WAVE3 in the MDA-MB-231 and MDA-MB-231/Adr cells after transfection. Compared with the control group, ^*∗∗*^*P* < 0.01.

**Figure 4:**
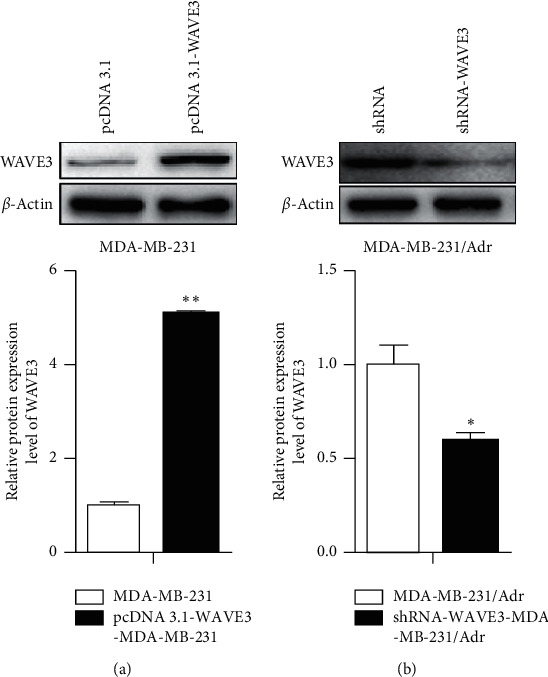
Protein expression level of WAVE3 in the MDA-MB-231 and MDA-MB-231/Adr cells after transfection. Compared with the control group, ^*∗*^*P* < 0.05 and ^*∗∗*^*P* < 0.01.

Results (Figure 3) showed that the mRNA and protein expression levels of WAVE3 increased significantly in the MDA-MB-231 cells transfected with pcDNA3.1-WAVE3 plasmid (*P* < 0.01), and Figure 4 shows that the levels decreased significantly in the MDA-MB-231/Adr cells with WAVE3 knocked out (*P* < 0.05 or *P* < 0.01).

### 3.4. Changes in Proliferation Activity of the MDA-MB-231 and MDA-MB-231/Adr Cells before and after Transfection with Plasmid with High WAVE3 Expression or shRNA

**Figure 5:**
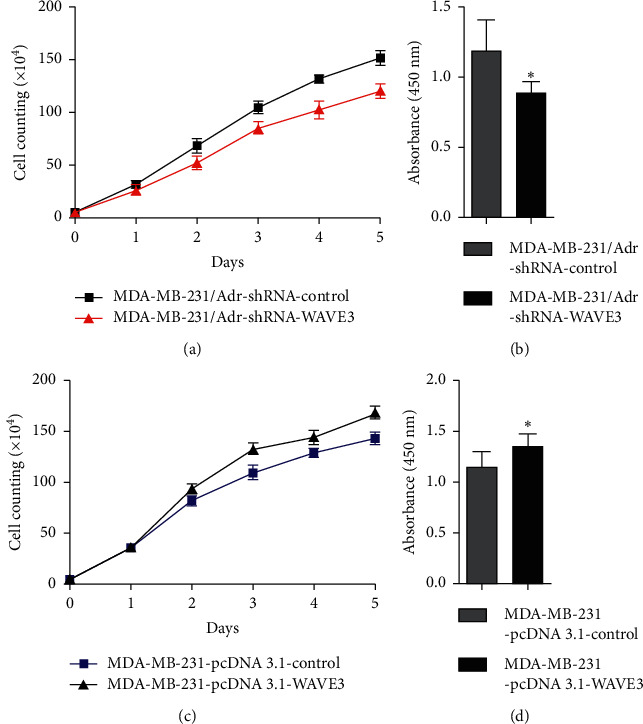
Changes of the number of the MDA-MB-231 and MDA-MB-231/Adr cells over time after transfection. Compared with the control group, ^*∗*^*P* < 0.05.

Results (Figure 5) showed that proliferation activity of the MDA-MB-231 cells increased significantly after transfection with pcDNA3.1-WAVE3 plasmid (*P* < 0.05), and it decreased significantly in the MDA-MB-231/Adr cells with WAVE3 knocked out (*P* < 0.05).

### 3.5. Changes in Invasion Ability of the MDA-MB-231 and MDA-MB-231/Adr Cells before and after Transfection with Plasmid with High WAVE3 Expression or shRNA

**Figure 6:**
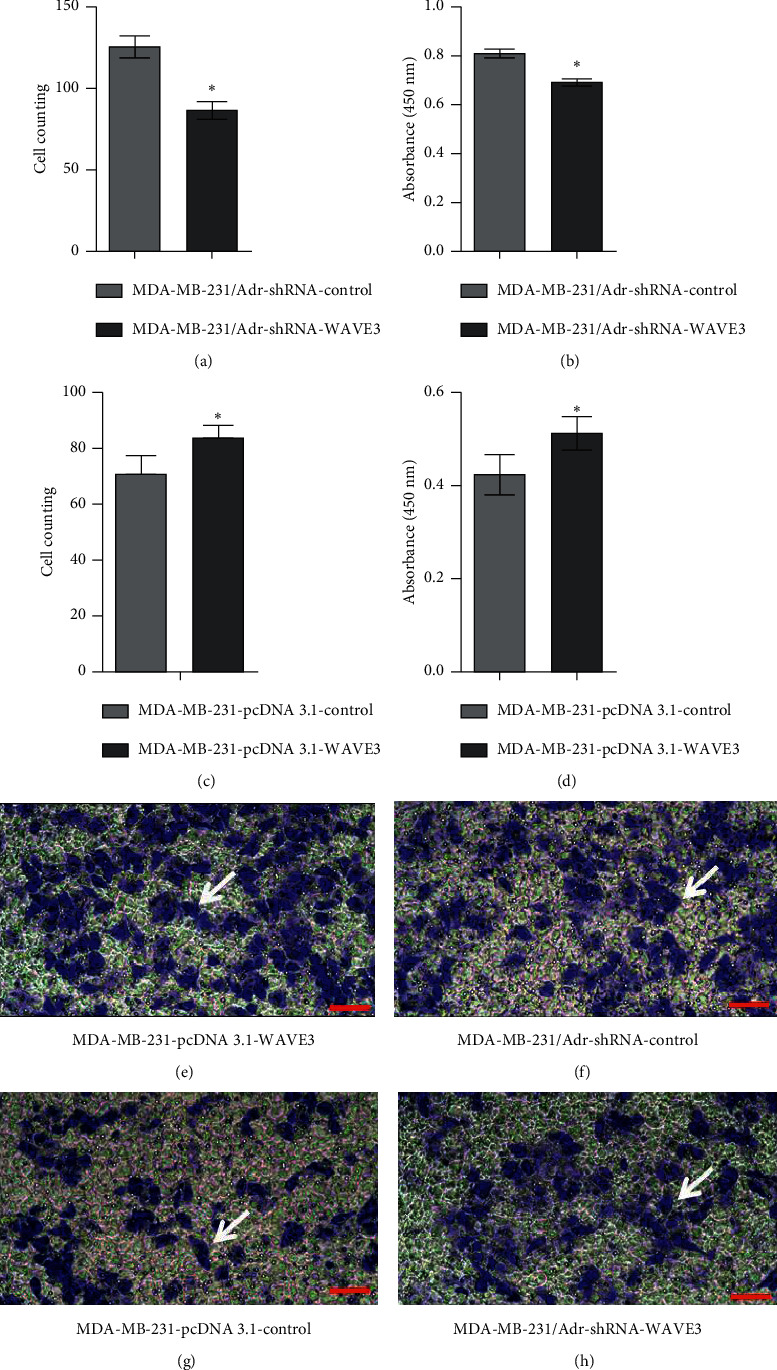
Changes in invasion ability of the MDA-MB-231 and MDA-MB-231/Adr cells after transfection. Compared with the control group, ^*∗*^*P* < 0.05 and ^*∗∗*^*P* < 0.01. The magnification of the photomicrograph is 300 times.

Results (Figure 6) showed that invasion ability of the MDA-MB-231 cells increased significantly after transfection with pcDNA3.1-WAVE3 plasmid, and it decreased significantly in the MDA-MB-231/Adr cells with WAVE3 knocked out.

### 3.6. Effect of FZYLF on In Vitro Proliferation Activity of Human Breast Cancer Cells before and after WAVE3 Gene Silencing in Human Breast Cancer Cell Lines

**Figure 7:**
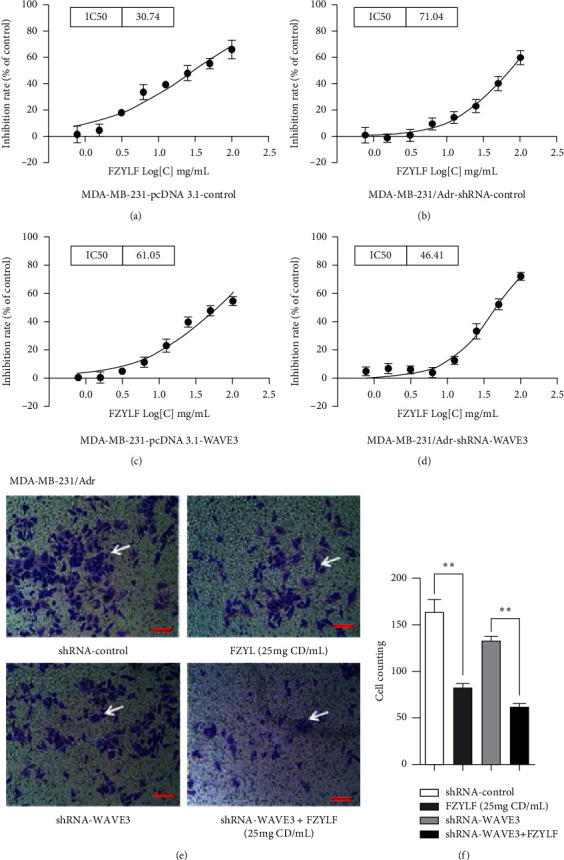
Effect of FZYLF on in vitro invasion ability of the drug-resistant human breast cancer cells before and after WAVE3 gene silencing. Compared with the control group, ^*∗*^*P* < 0.05 and ^*∗∗*^*P* < 0.01. The magnification of the photomicrograph is 300 times.

Results (Figure 7) showed that the MDA-MB-231 cells transfected with pcDNA3.1-WAVE3 plasmid had an increased resistance to FZYLF, with increase of IC_50_ from 30.74 mg/mL to 61.05 mg/mL, and the MDA-MB-231/Adr cells with WAVE3 knocked out had significantly increased sensitivity to FZYLF, with decrease in IC_50_ from 71.04 mg/mL to 46.41 mg/mL (*P* < 0.05 or *P* < 0.01). FZYLF could reduce the invasion ability of the MDA-MB-231 and MDA-MB-231/Adr cells, and its intervention with the expression level of WAVE3 also had an effect on the invasion ability of the cells (*P* < 0.05 or *P* < 0.01).

### 3.7. Effect of FZYLF on Protein Expression of WAVE3, I*κ*B*α*, NF-*κ*B, and MMP-9 in Human Breast Cancer Cells before and after WAVE3 Gene Silencing in Drug-Resistant Human Breast Cancer Cell Lines

**Figure 8:**
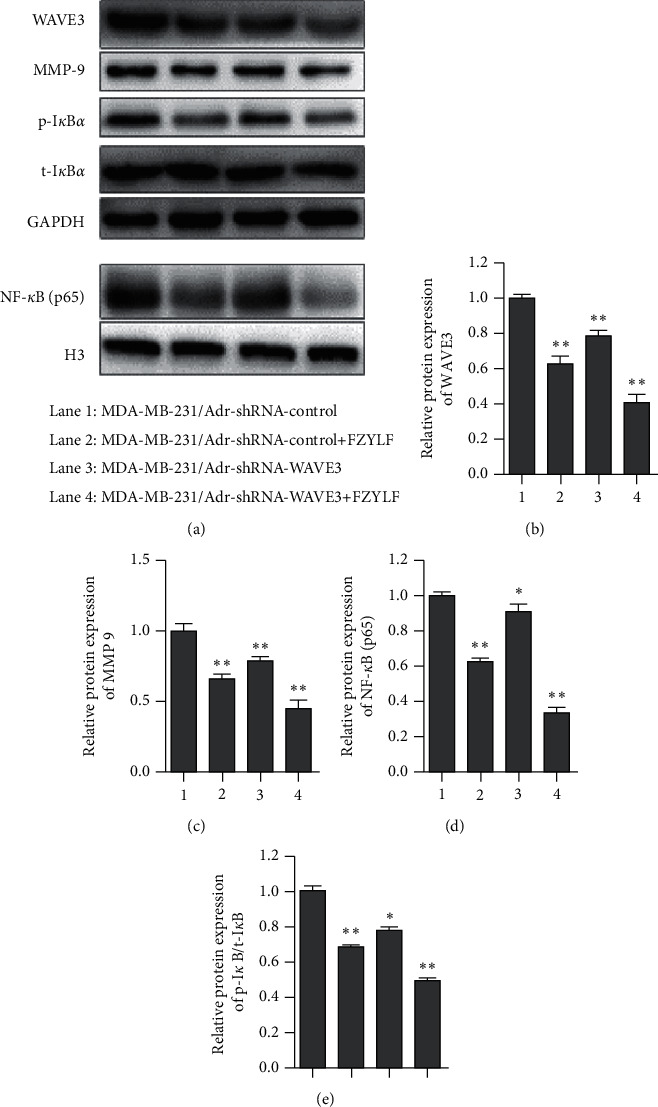
Effect of FZYLF on protein expression of WAVE3, I*κ*B*α*, NF-*κ*B, and MMP-9 in human breast cancer cells before and after WAVE3 gene silencing (FZYLF 20 mg crude drugs/mL). Compared with the control group, ^*∗*^*P* < 0.05 and ^*∗∗*^*P* < 0.01.

Results (Figure 8) showed that the MDA-MB-231/Adr cells with WAVE3 knocked out were more sensitive to regulation by FZYLF, which could significantly inhibit the protein expression levels of WAVE3, MMP-9, p-I*κ*B*α,* and NF-*κ*B (p65) (*P* < 0.05 or *P* < 0.01), but FZYLF had no significant effect on the protein expression level of t-I*κ*B*α*. GAPDH was the internal reference for cytoplasmic proteins and H3 for nuclear proteins.

### 3.8. Effect of FZYLF on MDA-MB-231/Adr and MDA-MB-231/Adr-siRNA WAVE3 Xenografted Tumor

**Figure 9:**
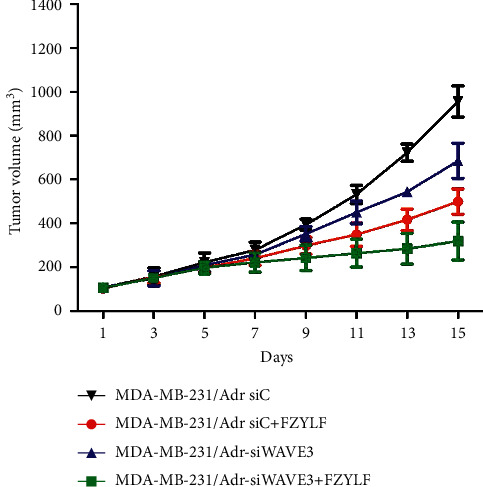
Effect of FZYLF on growth volume of xenografted tumor of the adriamycin-resistant human breast cancer cell line MDA-MB-231/Adr in nude mice.

**Figure 10:**
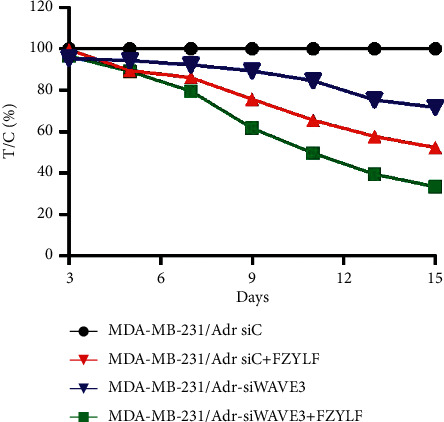
Effect of FZYLF on relative proliferation rate of xenografted tumor of adriamycin-resistant human breast cancer cell lines in nude mice.

**Figure 11:**
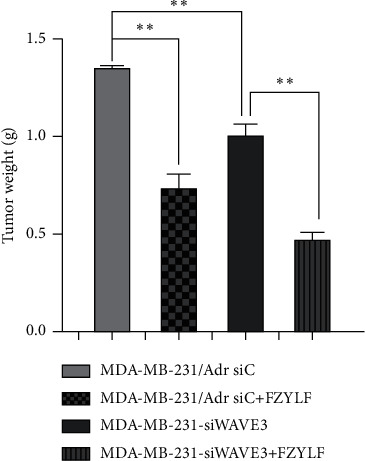
Effect of FZYLF on tumor weight of xenografted tumor of adriamycin-resistant human breast cancer cell lines in nude mice. Compared with the MDA-MB-231/Adr-siC group, ^*∗*^*P* < 0.05 and ^*∗∗*^*P* < 0.01.

**Figure 12:**
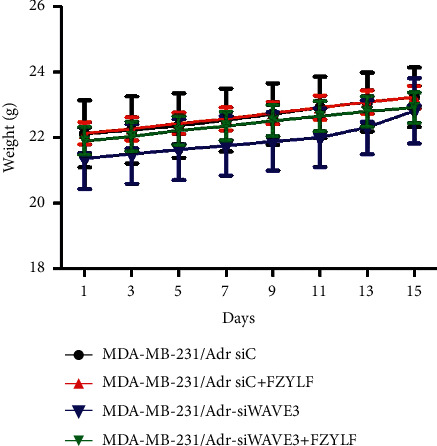
Effect of FZYLF on body weight of the nude mice bearing xenografted tumor of adriamycin-resistant human breast cancer cell lines.

**Figure 13:**
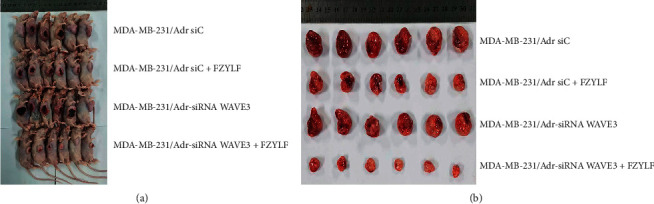
Tumor-bearing nude mice in each group and size of tumor.

The cell lines MDA-MB-231/Adr-siC and MDA-MB-231/Adr-siWAVE3 with low WAVE3 expression were inoculated into nude mice under the armpit, and the test article FZYLF (13 g crude drugs) was administered by gavage for 15 days consecutively. Results (Figures 9–13) showed that at day 9 of administration, FZYLF significantly reduced the tumor volume compared to the volume of the MDA-MB-231/Adr-siC xenografted tumor (*P* < 0.05). At day 15, compared to the MDA-MB-231/Adr-siC xenografted tumor group, T/C (%) was 52.29%, 71.74%, and 33.40% in the MDA-MB-231/Adr-siC + FZYLF, MDA-MB-231/Adr-siWAVE3, and MDA-MB-231/Adr-siWAVE3+FZYLF groups, respectively, and the tumor inhibition rate was 41.92%, 23.81%, and 64.87% in the MDA-MB-231/Adr + FZYLF, MDA-MB-231/Adr-siWAVE3, and MDA-MB-231/Adr-siWAVE3+FZYLF groups, respectively. FZYLF (13 g crude drugs/kg) had a T/C (%) of 62.06% and a tumor inhibition rate of 39.64% for MDA-MB-231/Adr xenografted tumor in nude mice.

## 4. Discussion

As a highly heterogeneous tumor, breast cancer is a heterogeneous disease composed of different solid tissues with different gene expression patterns and its heterogeneity may be caused by different types of gene mutation of normal epithelial cells of the breast that result in different groups of tumor cells. Through analysis of the molecular markers by immunohistochemistry, breast cancer is classified into four types based on different expressions of estrogen receptor (ER), progesterone receptor (PR), human epidermal growth factor receptor-2 (HER-2), and Ki67 index [19–21]. In TNBC, there is the absence of expression of the three receptors ER, PR, and HER-2, so TNBC is not sensitive to standard targeted therapies against breast cancer. Breast cancer of different types has different histopathological, molecular, and clinical features and requires different treatment methods. In Western countries, patients with TNBC account for 12∼17% of those with breast cancer, and the incidence rate of TNBC in Asian women is quite similar to that in white women. TNBC has the clinical features of high invasiveness, common local recurrence, and distant metastasis, and it has an obviously poor prognosis compared to the other types of breast cancer [22–24]. Currently, radiotherapy and chemotherapy are still the main treatments for breast cancer. High risk of relapse and metastasis and high mortality of TNBC require more research to improve the prognosis of this special type of breast cancer. Multidrug resistance of tumor is the main cause of chemotherapy failure, and the main mechanism of MDR involves reduced cellular uptake of drugs, increased cellular efflux of drugs, changes in apoptosis-related pathways, changes in drug-targeting molecules, enhanced DNA repair mechanism, increased activity of drug-metabolizing enzymes, etc. [25]. Therefore, it is important to further understand the processes of invasion and metastasis and find out the mechanism of action in invasion and metastasis of multidrug-resistant human breast cancer cells.

WAVE3 is a new actin-regulatory protein and its absence causes cellular abnormality in structure, migration, and invasion [26]. The NF-*κ*B pathway is an important mechanism in WAVE3-mediated cell migration, and Teng et al. [27] have found out that WAVE3 gene knockout in breast cancer cells can lead to decease in MMP-9 and inhibit the activation and nuclear transport of NF-*κ*B. The expression of WAVE3 is positively correlated with the extent of tumor invasion and progression and negatively correlated with clinic pathological parameters of tumor [6].

Breast cancer metastasis causes high economic burden to patients. Metastasis is an intrinsic feature of biological behavior of malignant tumor and also one of the root causes of treatment failure. TCM has unique advantages in the prevention of tumor recurrence and metastasis and the development of clinically effective Chinese compound formulas provides support for solving this clinical problem. According to TCM, “deficiency of healthy qi” is the ultimate cause of occurrence and development of tumor and it is present through the course of disease. Insufficiency of healthy qi is the pathological basis of tumor occurrence and deficiency is the root cause of disease. Reinforcing healthy qi is an essential approach in tumor treatment. Many studies have demonstrated that this treatment approach can not only improve the quality of life of patients with malignant tumor and reduce the adverse reactions of radiotherapy and chemotherapy but also optimize the therapeutic effect of chemotherapy through combined use with chemotherapy, and this approach can also prolong the progression-free survival and overall survival of patients [28–30]. FZYLF is a prescription used commonly in our department to treat deficiency of both qi and yin after breast cancer surgery and radiotherapy and chemotherapy. Breast cancer is a disease of root (actual) deficiency and branch (seeming) excess, or specifically, deficiency resulted from deficiency and concurrent deficiency and excess. Root deficiency occurs mainly in the liver, spleen, and kidney, and branch excess is mainly qi stagnation, blood stasis, phlegm turbidity, and heat toxin. Internal deficiency of healthy qi and yin-yang disharmony in Zang-Fu organs are the basis and the seven emotions and internal damage are the important factors in occurrence of breast cancer. FZYLF improves the quality of life of breast cancer patients and reduces the adverse reactions of surgery, chemotherapy, and radiotherapy.

WAVE3 had different expression levels in different drug-resistant breast cancer cells: the expression of WAVE3 was higher in the drug-resistant MDA-MB-231/Adr and MDA-MB-231/Taxol cells than in the nondrug-resistant MDA-MB-231 cells, and it was higher in the drug-resistant MCF-7/Adr and MCF-7/Taxol cells than in the nondrug-resistant MCF-7 cells. The expression of WAVE3 was the highest in the MDA-MB-231/Adr cells. It was indicated in the cell transfection experiment in the present study that the mRNA and protein expression levels of WAVE3 increased significantly in the MDA-MB-231 cells transfected with pcDNA3.1-WAVE3 plasmid, and the levels decreased significantly in the MDA-MB-231/Adr cells with WAVE3 knocked out. Experiments funded by the National Natural Science Foundation of China (NSFC) and conducted in our hospital have demonstrated that the expression level of WAVE3 increases significantly in the tissues of breast carcinoma *in situ* and the expression level of WAVE3 is also higher in the tissues of invasive breast carcinoma than in those of breast carcinoma *in situ*. These results suggest that WAVE3 is correlated to the proliferation and invasion of breast cancer. This correlation is confirmed clinically and cellularly.

In the in vitro experiment, intervention with FZYLF reduced the invasion ability of the MDA-MB-231 cells transfected with pcDNA3.1-WAVE3 plasmid and the MDA-MB-231/Adr cells with WAVE3 knocked out. In the in vivo experiment, FZYLF (13 g crude drugs/kg) showed a T/C (%) of 62.06% and a tumor inhibition rate of 39.64% for the MDA-MB-231/Adr xenografted tumor in nude mice and could significantly inhibit the growth of the xenografted tumor. It can be seen from the changes in tumor weight and growth volume of the drug-resistant human breast cancer cell line MDA-MB-231/Adr xenografted tumor in nude mice that FZYLF inhibits the proliferation and metastasis of the MDA-MB-231/Adr cells.

It was demonstrated in the mechanism study that FZYLF could significantly inhibit the protein expression levels of WAVE3, MMP-9, p-I*κ*B*α,* and NF-*κ*B (p65), but it had no significant effect on the protein expression level of t-IkBa. So, it is deduced that FZYLF may inhibit the MDA-MB-231/Adr cells through regulation of WAVE3, by influencing the protein expression of MMP-9, p-I*κ*B*α,* and NF-*κ*B (p65). MMP-9, p-I*κ*B*α,* and NF-*κ*B (p65) have a certain relationship with the invasion and metastasis of breast cancer, and further study is needed to find out the pathway(s) through which they work.

## 5. Conclusion

This study explores the mechanism of action of FZYLF in regulation of the invasion and metastasis of the MDA-MB-231/Adr human breast cancer cells through WAVE3. The effect of FZYLF on proliferation and invasion of the cells before and after WAVE3 knockout is investigated by Transwell assay, PCR, WB, and flow cytometry. The antitumor activity of FZYLF is studied by establishing a xenografted tumor in nude mice. It is revealed that FZYLF significantly inhibits the invasion and metastasis of the multidrug-resistant breast cancer cells MDA-MB-231/Adr and the mechanism of action may be related to its inhibition of the abnormal protein expression of WAVE3 gene, MMP-9, p-I*κ*B*α,* and NF-*κ*B (p65). That FZYLF regulating the invasion and metastasis of human breast cancer cells through WAVE3 is a complex process which involves many proteins and pathways, the specific links in mediation of cell metastasis mechanism by FZYLF are still unclear, and further experiment is needed in the future to verify the effect of FZYLF and the related mechanism.

## Figures and Tables

**Table 1:** List of instruments.

Instrument	Purchased from	Model
Biological safety cabinet	Suzhou Purification Equipment Co., Ltd.	BHC-1300A/B2
Carbon dioxide incubator	SANYO Japan	MCO-15AC
Fluorescence inverted biomicroscope	Nanjing Jiangnan Novel Optics Co., Ltd.	XD-202
Tabletop high-speed centrifuge	SCILOGEX	D2012
Low-temperature high-speed centrifuge	SCILOGEX	D3024R
Microplate reader	Thermo Scientific	MUTISKAN MK3
Precision electronic balance	Satorius	BSA224S
Microvertical electrophoresis tank	Shanghai Tanon Technology Co., Ltd.	VE 180
Transfer electrophoresis cell	Shanghai Tanon Technology Co., Ltd.	VE 186
Electrophoresis apparatus	Shanghai Tanon Technology Co., Ltd.	EPS 300
Decolorizing shaker	Jiangsu Jintan Ronghua Instrument Manufacturing Co., Ltd.	TY-80B
Mute mixer	Jiangsu Haimen Qilin Beier Instrument Manufacturing Co., Ltd.	WH-986

## Data Availability

All data used during the study are available from the corresponding author.

## Supplementary Materials

Supplementary Materials Quality control of Fuzheng Yiliu formula . Figure S1: HPLC chromatogram of asiatic acid reference substance. Figure S2: HPLC chromatogram of Fuzheng Yiliu formula. Figure S3: HPLC chromatogram of Maorenshen (*Actinidia valvata* Dunn, MRS) negative sample.
