# Supplementary material for: Fuzheng Yiliu Formula Regulates Tumor Invasion and Metastasis through Inhibition of WAVE3 Expression
Source: Evid Based Complement Alternat Med. 2021 Mar 27;2021:8898668. doi: 10.1155/2021/8898668 (PMC8019629; doi:10.1155/2021/8898668)
Supplement: Supplementary Materials — Quality control of Fuzheng Yiliu formula . Figure S1: HPLC chromatogram of asiatic acid reference substance. Figure S2: HPLC chromatogram of Fuzheng Yiliu formula. Figure S3: HPLC chromatogram of Maorenshen (Actinidia valvata Dunn, MRS) negative sample. [file 8898668.f1.pdf]

Supplementary materials

Fuzheng Yiliu Formula Quality Control

In this study, the method of extracting Asiatic acid from Maorenshen (*Actinidia valvata Dunn*, MRS) in Fuzheng Yiliu Formula was used for experimental research and HPLC method was used for chromatographic determination. The results show that the HPLC method for content determination is simple, accurate and reliable, and can be used for the quality control of Fuzheng Yiliu Formula.

1. Instruments and reagents

Water 1525 high performance liquid chromatograph, Waters 2489 UV detector; triethylamine (Sinopharm Chemical Reagent Co., Ltd.) methanol; acetonitrile (chromatographically pure, purchased from Merck Co., Ltd., Germany); ultrapure water.

2. Chromatographic conditions

Chromatographic column AgilentHC-C18 column, (4.6mm×250mm, 5.0μm), mobile phase: acetonitrile (30mmol/L)-ultrapure water (38:62); flow rate: 1.0ml/min, column temperature 25℃, UV detection wavelength 210nm, injection volume 20μl, running time 40min. Mobile phase: acetonitrile (30mmol/L)-ultrapure water (38:62). The injection volume is 20ul

3. Extraction of test samples

Dangshen (*Radix Codonopsis*, DS) 7.5g, Maidong (*Radix Ophiopogonis*, MD) 5g, Maorenshen (*Actinidia valvata Dunn*, MRS) 7.5g, Shijianchuan (*Salviae Chinensis Herba*, SJC) 7.5g, stir-fried Biejia (*Carapax Trionycis*, BJ) 7.5g and crude Yiyiren (*Semen Coicis*, YYR) 7.5g ，were weighed, placed in a 1000ml round bottom flask, and added 10 times the amount of water (500ml), soaked for 1 hour, heated reflux for extraction for 2 hours, collected the extract, heated and concentrated to 1g crude drug/mL (50ml), stored at 4 ℃ for later use.

4. Extraction of negative samples of Maorenshen (*Actinidia valvata Dunn*, MRS)

Dangshen (*Radix Codonopsis*, DS) 7.5g, Maidong (*Radix Ophiopogonis*, MD) 5g, Shijianchuan (*Salviae Chinensis Herba*, SJC) 7.5g, stir-fried Biejia (*Carapax

Trionycis, BJ) 7.5g and crude Yiyiren (*Semen Coicis*, YYR) 7.5g ， were weighed, placed in a 1000ml round bottom flask, and added 10 times the amount of water (500ml), soaked for 1 hour, heated reflux for extraction for 2 hours, collected the extract, heated and concentrated to 1g crude drug/mL (50ml), stored at 4 ℃ for later use.

5. Preparation of test sample solution

5ml of the above-mentioned concentrated and standby test sample was pipetted, transferred to a 10ml centrifuge tube, added 5ml of methanol, mixed, filtered with a 0.45μm organic filter membrane, and the subsequent filtrate was taken for testing The product solution was obtained.

6. Preparation of Maorenshen (*Actinidia valvata Dunn*, MRS) negative sample solution

5ml of the concentrated negative Maorenshen (*Actinidia valvata Dunn*, MRS) sample was pipetted,, transferred to a 10ml centrifuge tube, 5ml of methanol was added, mixed well, filtered with a 0.45μm organic filter membrane, the filtrate was taken, and the test solution was obtained.

7. Preparation of reference substance stock solution

1.1 mg of the asiatic acid reference substance was accurately weighed, placed in a 5ml centrifuge tube, dissolved in methanol and adjusted to the mark to obtain a reference substance stock solution with a concentration of asiatic acid 0.22mg/ml.

8. Determination method

20 μL each of the reference substance solution and the test solution solution are accurately drawn and injected into the liquid chromatograph for determination

9. Results

The chromatographic separation of the asiatic acid reference substance is good, and the results are shown in Figure 1. The chromatographic separation of asiatic acid in the test solution is good, and the results are shown in Figure 2.

For the negative control solution of Maorenshen (*Actinidia valvata Dunn*, MRS), no peak appears at the corresponding position of Asiatic acid in the chromatogram. The result is shown in Figure 3.

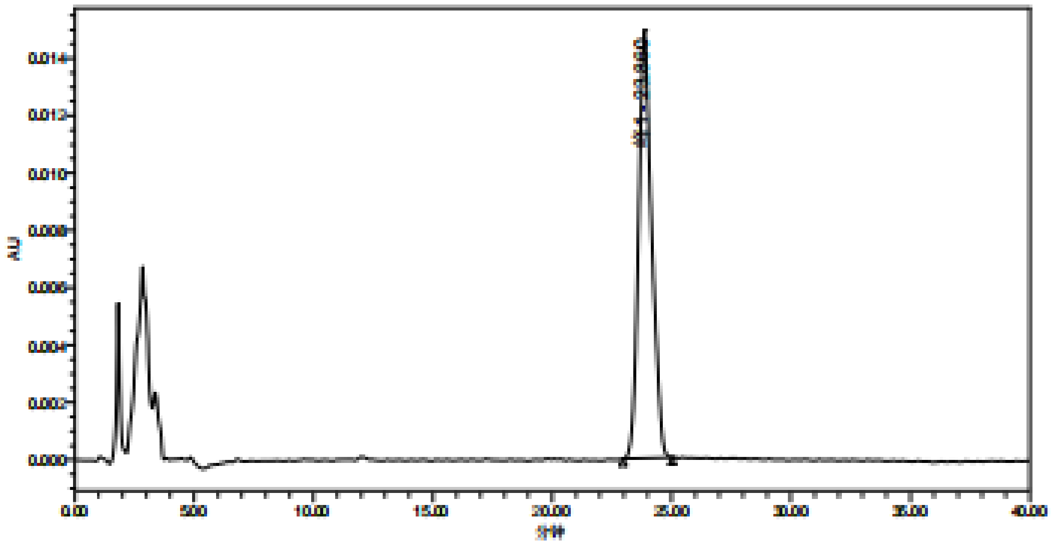

Figure 1 HPLC chromatogram of Asiatic acid reference substance

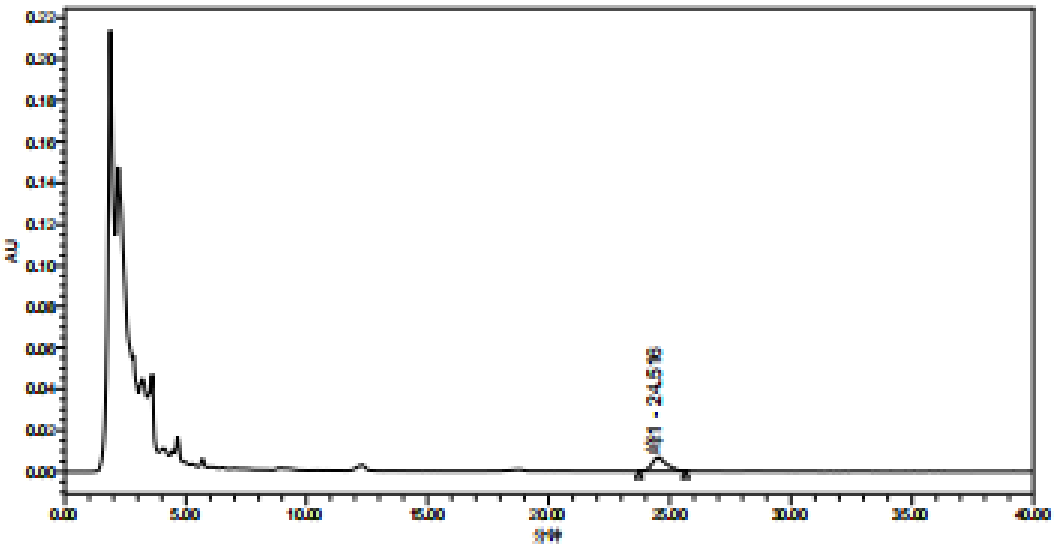

Figure 2 HPLC chromatogram of Fuzheng Yiliu Formula

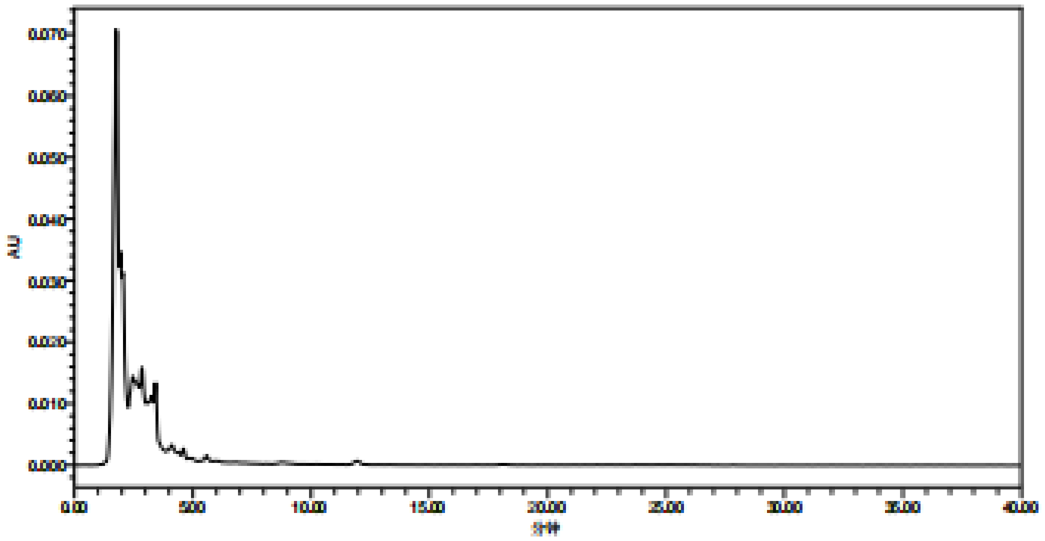

Figure 3 HPLC chromatogram of Maorenshen (*Actinidia valvata Dunn, MRS*) negative sample
